# Towards a new generation of agricultural system data, models and knowledge products: Information and communication technology

**DOI:** 10.1016/j.agsy.2016.09.017

**Published:** 2017-07

**Authors:** Sander J.C. Janssen, Cheryl H. Porter, Andrew D. Moore, Ioannis N. Athanasiadis, Ian Foster, James W. Jones, John M. Antle

**Affiliations:** a Wageningen University and Research Centre, Postbus 47, 6700AA Wageningen, Netherlands; b Agricultural & Biological Engineering, University of Florida, PO Box 110570, Gainesville, FL 32611, USA; c CSIRO Agriculture & Food, GPO Box 1600, Canberra, ACT, 2601, Australia; d Computation Institute, University of Chicago, IL, USA; e Oregon State University, Corvallis, OR, USA

**Keywords:** Agricultural models, ICT, Linked data, Big data, Open science, Sensing, Visualization

## Abstract

Agricultural modeling has long suffered from fragmentation in model implementation. Many models are developed, there is much redundancy, models are often poorly coupled, model component re-use is rare, and it is frequently difficult to apply models to generate real solutions for the agricultural sector. To improve this situation, we argue that an open, self-sustained, and committed community is required to co-develop agricultural models and associated data and tools as a common resource. Such a community can benefit from recent developments in information and communications technology (ICT). We examine how such developments can be leveraged to design and implement the next generation of data, models, and decision support tools for agricultural production systems. Our objective is to assess relevant technologies for their maturity, expected development, and potential to benefit the agricultural modeling community. The technologies considered encompass methods for collaborative development and for involving stakeholders and users in development in a transdisciplinary manner.

Our qualitative evaluation suggests that as an overall research challenge, the interoperability of data sources, modular granular open models, reference data sets for applications and specific user requirements analysis methodologies need to be addressed to allow agricultural modeling to enter in the big data era. This will enable much higher analytical capacities and the integrated use of new data sources. Overall agricultural systems modeling needs to rapidly adopt and absorb state-of-the-art data and ICT technologies with a focus on the needs of beneficiaries and on facilitating those who develop applications of their models. This adoption requires the widespread uptake of a set of best practices as standard operating procedures.

## Highlights

• Next generation agricultural models could benefit from recent developments in information and communications technology and data
• Integrated development of modeling technologies, ICT infrastructures and user interaction is highlighted through use cases
• Interoperability of data, granular models, reference data sets and requirements analysis are identified research challenges.

## 1 Introduction

Information and computer technology (ICT) is changing at a rapid pace. Digital technologies allow people to connect across the globe at high speeds at any time (Gartner, 2016). Even those in remote, developing regions increasingly have the ability to connect online via telephone and Internet providers (Danes et al., 2014). Satellite and drone capabilities can provide remotely sensed data in real-time regarding in-season crop growth and development, soil moisture, and other dynamic variables (e.g. Capolupo et al., 2015). High performance computing can be used to process large amounts of data in a short time frame, to make sense of large quantities of structured and unstructured data (i.e. “big data”; NESSI, 2012) collected using new sensing technologies, and to scale and validate models in ways not previously possible. Web and cloud technologies permit these capabilities to be made available to large numbers of end users with a convenience and cost that was previously inconceivable (Foster, 2015). As a result of these and other developments, society expects more and higher-quality information to be available in support of daily decision-making.

Our enthusiasm for these new technologies in the agricultural sciences must be tempered by a realization that our modeling and decision support systems have not kept up with technology. Indeed, many frameworks used in these systems date back to the 1970s through the 1990s, prior to the availability of today's advanced data collection, computing, storage, access, processing technologies, software languages and coding standards. Thus, we see two distinct opportunities for applying modern ICT to agricultural systems modeling. First, advances such as big data, crowdsourcing (i.e. sourcing data and information through distributed networks of respondents), remote sensing, and high performance computing can be used to advance the *science* of agricultural systems modeling. Second, new technologies can be used to transform the *practice and application* of agricultural systems modeling by making it far more collaborative, distributed, flexible, and accessible. As clearly shown in a recent thematic issue of *Environmental Modeling and Software* (Holzworth et al., 2014a, Athanasiadis et al., 2015), the *science* of agricultural systems modeling is progressing steadily and adopting various new ICT technologies to advance the science on a case-by-case basis. However, *the practice and application* of agricultural systems modeling is not progressing as fast, leading to lack of applications using agricultural systems models. Thus, an important feedback from *application* to *science* is absent and needs to be established, as also discussed by Holzworth et al. (2015) for cropping systems models. The focus of this review is thus not on relevant ICT technologies for the modeling scientist working at a university or research institute, but on ways to facilitate the involvement of actors beyond the academy. As discussed in the companion article by Antle et al. (this issue), the result of achieving such involvement will be a “next generation” modeling community (NextGen) that includes not only modelers and model developers working across disciplines, spatial scales, and temporal scales to exploit new data sources and to produce and apply new models, but also software developers to produce the NextGen modeling frameworks, data processing applications, and visualization tools.

In this paper, we approach the envisioned NextGen of agricultural models and the supporting modeling community from the ICT perspective. Our objective is to assess relevant technologies for their maturity, expected development, and potential to benefit the agricultural modeling community. The technologies considered encompass methods for collaborative development and for involving stakeholders and users in development in a transdisciplinary manner.

We assess recent ICT developments through five use cases that have been formulated to support the vision for a NextGen modeling community (see also the introductory overview by Antle et al. (submitted, a) and accompanying papers by Jones et al. (2017) and Antle et al. (2017)).

**Fig. 1:**
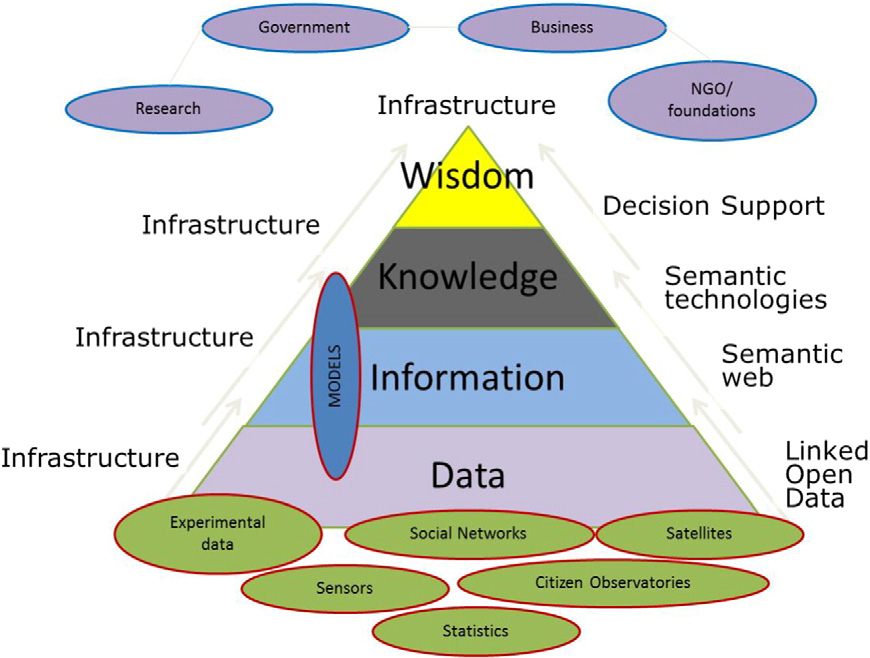
Knowledge Pyramid linking data to information to knowledge and wisdom, in which data is the raw material for the development of applications addressing decision making through wisdom in research, government, business and ngo/foundations (adapted from Lokers et al., 2016).

**Fig. 2:**
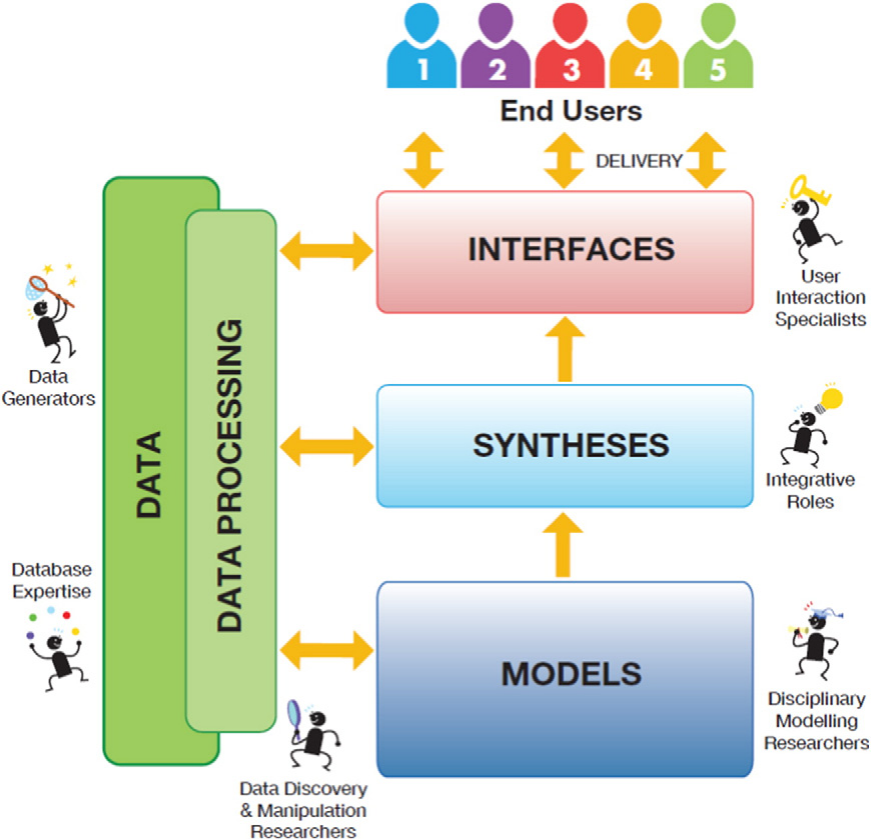
Application chains describing the flow of data and information through layers of modeling, syntheses and interfacing towards end-users with the role of different actors along the information chain.

A NextGen of applications based on agricultural systems modeling can help companies, governments, and farmers in the food chain to make informed decisions. The concepts of knowledge chain (Fig. 1) and application chains (Fig. 2) provide complementary perspectives on the value and positioning of modeling in the broader context of decision making and ICT, and are used to loosely organize the content of this paper. A **knowledge chain** is a set of linked steps by which data are processed into information, knowledge and finally wisdom as used in decision making. This perspective postulates that data comprise a raw material that, when combined with description and quality attributes, leads to information. Information can be linked to other information sources and placed in causal chains to produce knowledge. Ultimately, knowledge serves as an input for decisions based on wisdom, which cannot be digitized and which exists in the mind of a decision-maker. A second perspective focuses on **application chains**. Agricultural models must be engaged in an infrastructure consisting of both software (e.g. in layers of data access, processing, analysis and visualization) and hardware (i.e., servers, computing capacity, and storage) as depicted in Fig. 2. Based on the data in the infrastructure, applications targeted at end-users serve information and knowledge, e.g. a yield forecast to a supply chain manager; effects on farm income of a policy change; estimates of disease related crop damage to a farmer. Application chains may be simple or complex, and may include, for example, data access, extraction, transformation (e.g. summarization or interpolation), and integration operations; one or multiple models; integration of output from different models; and model output transformation, analysis, and visualization steps. Design of the application chains must consider not only the end-users, but the full spectrum of users of NextGen ICT infrastructure including primary data collectors, database professionals, software developers, modelers and the end-users of knowledge and information.

This paper is organized according to the different layers of the knowledge chain and the covering actors and elements of the application chains, focused on the NextGen of agricultural systems model applications. Section 2 introduces the users and the use cases where NextGen models could potentially play a large role. Section 3 describes the actors active along the application chain. 4, 5 cover developments in data as the raw material of any modeling effort and new technologies that can assist with the presentation of modeling analyses to users. In Section 6, we discuss how agricultural models (in the strict sense) will need to be developed and implemented to enhance their usefulness in concert with parallel ICT advances, and in Section 7 we discuss the importance of new interfaces from software to users and between software artifacts. Finally, in Section 8 we make a set of recommendations for steps that need to be taken to enable knowledge and application chains to be created for the next generation of agricultural knowledge products.

## 2 Envisioning future uses of NextGen agricultural models

### 2.1 Reference use cases

Numerous use cases can be developed to represent the stakeholders who define the outputs and characteristics of NextGen agricultural modeling applications. Five such use cases have been used to determine the most relevant ICT technologies to discuss in further detail as part of this paper; they have been described more fully in the introductory paper (Antle et al., submitted, a). The use cases are:

1. A farm extension officer in Africa who uses a decision support tool based on research results and modeling tools;
2. A researcher at an international agricultural research station, who tries to develop new technologies for sustainable intensification and wants to assess different technology options.
3. An investment manager at a donor organization who seeks to evaluate different project proposals to identify the projects most likely to be effective.
4. A farm advisor in precision agriculture who assists farmers in using high tech data streams through farm management software.
5. A consultant to companies in the food supply chain who uses web-based tools to evaluate the footprint of products and value chains

The use cases have been chosen to represent a wide range of farming systems, beneficiaries, and requirements for data and modeling components. Smallholder farming systems are featured in use cases 1, 2 and 3, as addressing the needs of these systems is considered to be essential to achieving food security in developing regions where smallholder farms account for most food production (Dixon et al., 2004). Note that a broad range of users was deliberately introduced into the use cases, in order to reveal the potential for developing the next generation of real-world model applications. The typical situation when thinking about ICT for agricultural modeling has previously been to consider mainly one user type only, that of the researcher/scientist.

In this paper the 5 use cases have been analyzed from an ICT perspective and the deficiencies of current agricultural modeling systems for addressing each use case have been identified. We have then proposed, in narrative form, a draft application chain for each use case that would remedy these deficiencies.(i.e., a simple draft proposal intended to generate discussion) Finally, an overall synthesis of these 5 ICT solutions is used to motivate the rest of the paper.

### 2.2 Use case 1 - farm extension in Africa

#### 2.2.1 Problem statement

Jan works as a farm extension officer in an area in southern Africa where many farms are extremely small, incomes are low, and farmers typically grow maize and beans as staple crops for their family's subsistence and to sell for cash. Some households may have livestock and/or grow vegetables. The aim of the extension service is to help farmers achieve higher and more stable yields of maize and also to advise them on improving their nutrition so that they obtain sufficient protein and micronutrients for healthy families. Jan obtains information on new varieties of maize and beans that are now available to farmers in the area. These new varieties are more drought and heat-tolerant, and the bean varieties are more resistant to a common foliar disease. Jan also has information on how to improve nutrient management of these crops using small doses of inorganic fertilizer along with animal manure and crop residues. He also has information on a new technique developed by CGIAR to partially harvest rainfall to increase water availability to the field and vegetable crops.

#### 2.2.2 Current deficiencies

Jan is not a modeler, but he can benefit from the outputs of agricultural production models and farm-scale economic models. In this case, the NextGen tool used by Jan must be able to combine or be combined with existing data about localized conditions (soils, weather, genetics, household economics, local markets, etc.) with farm-scale models to predict the viability of using the new varieties. These data are often difficult to access, if they exist at all. In many areas, weather data are considered to be proprietary and are not distributed freely. Good quality, localized soil data suitable for crop production modeling are usually non-existent or available only at a scale that is not practical at a field level. Information about household demographics and economics is rarely available except in cases where a research survey has been conducted recently in the area. In any case, these data may contain sensitive information, which should not be made publicly available until the data are anonymized. Pre-configured models appropriate to the smallholder systems of this region are needed, including components for mixed livestock/cropping systems driven by data relevant to management practices in common in the region (e.g., planting date “rules”, cropping densities, varieties cultivated, etc.).

The current infrastructure would not allow an easy answer for Jan. Existing models can simulate such systems, but the required data collection and model configuration would entail considerable efforts and direct collaboration with modelers and primary data collectors. Studies such as this, using current technology, would typically be performed to give generic suggested management for a region, with results not tailored to individual farms.

#### 2.2.3 A draft NextGen application chain

**Fig. 3:**
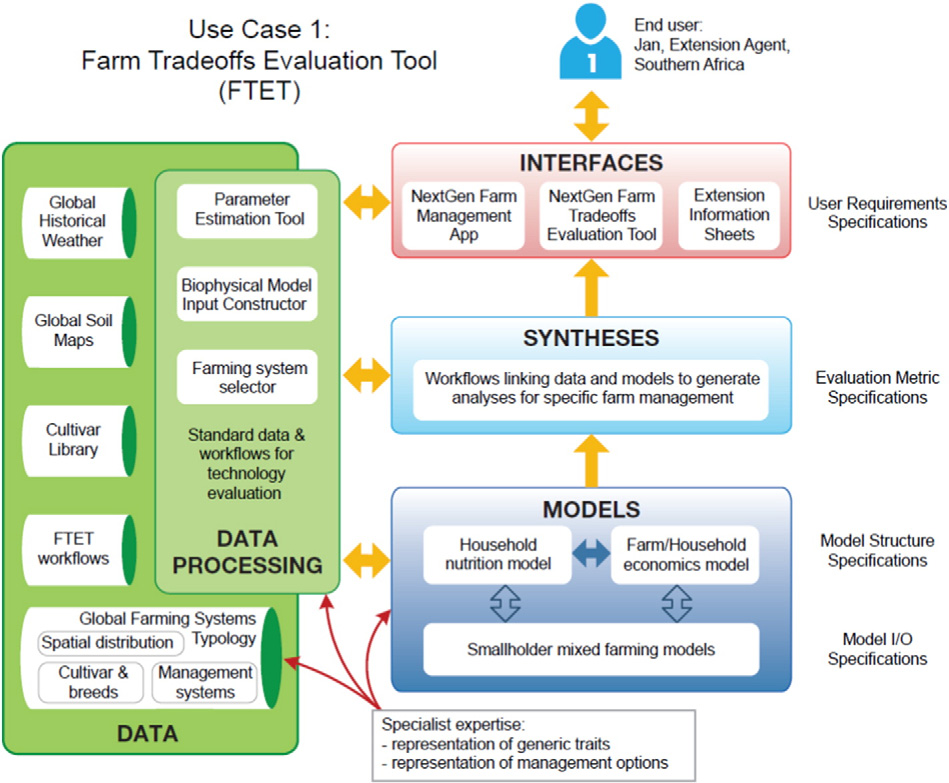
the components of the data, modeling and delivery infrastructure according to application chains to deliver use case 1, with as explanation: Jan has used the NextGen apps previously for evaluating improvements to cropping system management and so he is already familiar with the user interfaces and options available. He uses the **NextGen Farm Tradeoffs Evaluation Tool (FTET)** for use in evaluating the efficacy of the new varieties. The improved varieties of maize and beans have been developed by scientists at the CGIAR centers, who work closely with the **NextGen cultivar library** and have used the **NextGen parameter estimation tool** to develop crop model parameters for a **suite of NextGen models** for their new cultivars. These cultivar parameters are now stored in the cultivar library and are available for use in the NextGen suite of applications. Jan obtains information from the farmer and inputs these data into the **NextGen Farm Management App** on his smartphone, which has an interface developed specifically for the farming systems of his region. The app will help him determine combinations of system components that might best fit specific farm situations and register these management systems within the **Global Farming Systems Typology Database.** **Soil attribute and weather records** specific to the farm locations in Jan's region are already available in the NextGen database for use with the FTET. The FTET is a workflow that was generated for evaluating tradeoffs and synergies between management decisions and overall farm/household level profit and nutrition. Components of the tool include farm production using biophysical models, a nutritional analysis based on inputs and outputs to the farm, and prediction of household income under each scenario. Jan's input data from each household and the proposed improved varieties can be added to the workflow using the FTET user interface. Based on outputs from the FTET, Jan populates, distributes and discusses **extension information sheets** written in the local language that describe the components of crop and farming systems that are likely to succeed with the farm family.

Because farms vary in size, labor availability, soils, and other characteristics, Jan uses the NextGen tools to help tailor advice to each farm family that is practical, likely to be adopted, and provide the best outcome in terms of more stable production, higher income, and better nutrition. Jan obtains information from the farmer to input into his smart phone, which has NextGen apps that were developed for the farming systems of his region and that help him determine combinations of system components that might best fit specific farm situations. This software also provides template files for extension information sheets written in the local language(s) that describe the components of crop and farming systems that are likely to succeed with the farm family. The design-time narrative – shown graphically in Fig. 3 and its caption – a describes the components of an application chain could allow Jan to deliver the necessary information to the smallholder farmers that he serves.

### 2.3 Synthesis of the reference use cases

Our analysis of the five use cases concludes that they require a mix of recent innovations in both technology and data (Table 1). This overall analysis is necessarily qualitative and dependent in its details on the specifics of the 5 use cases and of our draft application chains; clearly the latter could have been developed in different ways. We see a clear emphasis on data integration in the broadest sense, with a need to deal with data from different domains and from different (and both private and public) sources. All use cases thus require a more intensive use and combination of data, which so far has not frequently occurred (Section 4). Only one of the 5 case studies (nr 3) relies on the synthesis of large masses of well-structured sensor data. In the other 4 cases, “big data” techniques will mainly be needed to synthesize multi-purpose databases such as farming systems typologies from diverse sources of relatively unstructured information. (See Section 5).

**Table 1:** Overall analysis of the five NextGen Use cases for their relevant IT and data aspects Scores are from ‘no score’; to + = element, but not crucial; to ++ = important innovation required; to +++ = crucial innovation required. Use cases are: Use case definitions: 1 = farm extension in Africa; 2 = developing technologies for sustainable intensification; 3 = investing in projects for sustainable intensification; 4 = management support for precision agriculture; 5 = supplying food products that meet corporate sustainability goals.

Characteristics	Use cases				
	1	2	3	4	5
**Users and usability**					
User identification (1)	+	++	+	+	++
Complexity (2)	++	+++	+++	++	++
User requirements (3)	++	++	++	+++	+++

**Data and IPR**					
Open data	+	+++	+++	+	+++
Private data	+++	+		+++	+++
Data integration	++	+++	+++	++	+++
‘New’ data sources (i.e., social media, remote sensing, crowdsourcing)	+++		+++	+++	
Big data			++	+	
Linked data and semantics		++	++	++	++

**Visualization**					
Targeted visualization required	+++	+	+++	+++	+++
Visual Analytics required			++	++	
Apps	+++		++	+++	+

**Model development**					
Model as components	+++	+++	+++	+++	+++
Model linking	+	+	++	++	++
Flexible workflow frameworks	++	+++	++	+	++
Collaborative development	++	++	+++	++	++

**IT infrastructure**					
Service-oriented architecture	+++	++	+++	+++	+++
Desktop based	partly	yes	no	no	yes
Application (app) based	yes	no	yes	yes	partly

(1) User identification refers to activities in which it is relatively unsure who the user really is, and this needs to be further investigated;

(2) Complexity is a subjective assessment of the overall complexity of the use case as judged from the number of data sources, ICT innovations and visualization techniques;

(3) User requirements refers to the extent to which additional user requirements analysis is needed to progress.

Most of the use cases depend on the availability of good quality, accessible and preferably open input data, suitable for use in modeling applications (Section 4). These data requirements seem to be the low-hanging fruit of any modeling system, but they are often surprisingly difficult to obtain in today's modeling world. Soil data, for example, are needed that have relevance to localized agricultural fields, are complete, and are suitable for use in crop models. The GlobalSoilMap program (Arrouays et al., 2104; globalsoilmap.org) will help relieve this constraint as it is completed, however, local data will be need to improve on these generic data sets to capture the heterogeneity.

Semantic web and linked data mechanisms can help to realize the use cases more easily and to enable data sharing across use cases, but they never appear to be crucial. Standardized data protocols, on the other hand, are vital: they will allow data to be shared, discovered, combined with other data from different sources, and used in multiple applications and analyses in various modeling domains. It seems likely that the use of Linked Open Data principles (see, Lokers et al., 2016) will ultimately be a central component of a NextGen framework (Section 4).

As a result of taking a use-case approach, the users of our draft application chains are already quite clearly identified; the main requirement for NextGen is to more explicitly define what these users really need through state-of-the-art requirements analysis techniques (Section 3). Each of the use cases formulates some general ideas and directions, but clearly much more information would be required to elaborate real applications.

Targeted visualization is needed to communicate results to users (Section 5). In one use case (#2), existing techniques could be used to visualize data in tables that are generated and analyzed for each realization. The other use cases require visualizations that are integrated into interfaces that are specific to the corresponding knowledge products, with a clear link to underlying data and assumptions. The means to efficiently design and generate such visualizations are not available in current tools. Interestingly, we identify that in some cases there are clear benefits from deploying knowledge through mobile and web channels rather than via desktop-based solutions.

Finally, in terms of modeling methods (Section 6), we see a clear to need to move from monolithic models to stable, robust, granular, and well-defined components. With the latter approach, models are no longer large containers of analytical steps, but instead services that can be driven flexibly by external programs. Models thus need to become advanced algorithms that can be called robustly in a service-oriented system (i.e. a set up in which an application can readily draw on a library of services available through online protocols for data access, processing and computation). From an infrastructure point of view, the availability of services for data and models (analytics) is crucial for realizing the service-oriented infrastructures underlying the applications.

Model linking and modeling frameworks play a role in some use cases, but to a lesser extent than a flexible environment in which a user can explore the potential of models (with the exception of Use Case 2). Once a workflow has been implemented, it is important that users be able to run the resulting configuration repeatedly in a stable environment. Methods such as virtual machines and containers (i.e. pre-configured set-up of an operating environment with installed programs that can be deployed flexibly on hardware) will likely play an important role in capturing such environments. A framework that allows researchers to generate and share such workflows, including connections to multiple data sources, will likely be a key element of the NextGen modeling infrastructure (Section 7).

## 3 Envisaged application chain users

### 3.1 Beneficiaries

Information and knowledge provided by NextGen applications can not only improve understanding but also change the balance of power, by allowing beneficiaries to better understand both the biological systems that they manage through their farming practices and each other's modes of operation. The rapidly increasing digitization of society along with the increasing availability of internet and mobile technologies in agricultural communities provides massive opportunities for the hitherto under-served (Danes et al., 2014). For example, farmers in Africa can now receive text messages regarding current crop prices, seed and fertilizer locations, and crop insurance, thus allowing them to make informed decisions based on up-to-date information (Rojas-Ruiz and Diofasi, 2014). NextGen applications need to break through from the scientist – end user dipole, and consult with a broad spectrum of stakeholders, including businesses, farmers, citizens, government, non-governmental organizations (NGOs), and research institutions. As noted in the introductory paper (Antle et al., submitted, a), NextGen model development starts with an understanding of information required by various stakeholders and then works back from those requirements to determine the models and data needed to deliver that information in the form that users want. ICT offers various techniques for scoping user requirements, from more traditional methods of user requirements analysis to modern techniques of user-centered design, in which software is built in direct contact with the end-user in short iterations. In the latter approach, user needs and requirements guide and modify the development in each iteration (Cockburn, 2006). To our knowledge, no application of user-centered design methodologies has been published in the scientific literature for agricultural models. Agricultural models – in contrast to some decision-support interfaces to existing models (McCown, 2002, Hochman et al., 2009) – were mostly developed with a push perspective in mind to expose the functionality and the rich data needs of the model, not to improve the usage of the end-user. This comes as a consequence of the way models have been historically developed: the primary user has always been the scientist. However, this will most likely not be the case for the NextGen of applications.

At the same time, opportunities to be in touch with end-users have become more numerous with the advent of mobile technology, social media, text messaging, radio and TV shows, and apps designed for tablets and mobile phones. At the moment there are still geographic areas where smallholder farmers lack the mobile networks for sharing data; however, access to SMS and voice message services is increasing rapidly and it is expected that end-users in rural areas in developing countries will skip the step of personal computers and make direct use of mobile phones and possibly tablets (Danes et al., 2014). This trend suggests that there is a huge untapped potential to boost the amount of information provided to farmers and processors in the chain, both because their needs are not yet defined and because services specifically focused on their use are not yet developed. Mobile technology offers a different user experience, as screens are smaller and handled under different circumstances. Thus mobile visualizations must be simple yet powerful if they are to motivate users to persist in their use. Often they only work with just a few data points, communicated to the end-user with stronger visual emphasis on color and readability.

### 3.2 Application chain developers

Data collectors, software and model developers, database experts, and user interaction specialists are needed to contribute new capabilities to the envisaged applications of NextGen agricultural models. Nonetheless, existing model development teams need to play a key role in defining the capabilities of future infrastructure oriented towards application development. Such interactions between model development teams and application developers can happen via existing agricultural modeling communities such as the Agricultural Model Intercomparison and Improvement Project (AgMIP; Rosenzweig et al., 2013) and MACSUR (*macsur.eu*) and by application oriented projects such as FACE-IT (*faceit-portal.org*, Montella et al. 2014), GeoShare (*geoshareproject.org*), and BIOMA (Donatelli and Rizzoli, 2008). Continued model development and application partnerships using collaborative design methodologies are a necessary component of successful development infrastructure (Holzworth et al., 2014a, Holzworth et al., 2014b).

A long-term strategy of the NextGen modeling system will need to be to entrain new model developers and other knowledge system specialists from the application community, especially in developing regions of the world, as also argued by Holzworth et al. (2015). Users and developers from emerging economies can bring unique perspectives that can guide the model development and application process to include key relevant components critical to their user communities. For example, a model developer in West Africa might emphasize the importance of soil phosphorus in yield limitations of that region – a modeling component lacking in many existing agricultural production models that were generated in regions where phosphorus is used at much higher input levels. Model users with deep knowledge of the decision-making requirements of emerging-economy actors, and of the ICT technologies available to them (e.g. mobile phones), can contribute to components that meet the unique needs of these actors.

Finally, software development is required to provide for access of NextGen data products and delivery of the final products to users. This top layer, represented in Fig. 2, is likely to include both proprietary products developed by private industry and non-proprietary products developed in the public sector. We envision that with the proper infrastructure, enabling rapid data discovery and use, the delivery of agricultural data products may become the realm of many small and medium-sized local enterprises that can profit from the opportunities provided by the data and products to develop mobile and web service applications for use directly by farmers and NGOs.

## 4 Agricultural data

### 4.1 Traditional data sources

We identify three main traditional methods of data collection of relevance for agricultural systems modeling:

1. Governments collect data for monitoring purposes, management of information and administrative procedures. These data, which include national statistics, weather data, monitoring data for subsidies and taxes, and data to monitor environmental performance, are generally uniform in format and are usually collected on a regularly scheduled basis for as long as they are relevant for policies.

2. Research projects collect data (e.g., field and household surveys, multi-dimensional panel data, soil sampling, measurements in laboratories) to meet specific project needs. These data are often incidental (i.e., collected on an irregular schedule) and not structured (i.e., non-uniform in format). In most cases, these data are not usable because they are not shared. We will discuss this point further in Section 4.4.

3. Industries (including farmers and business-to-business service operators) collect data for their own operations. They do not usually share data due to competitive or privacy concerns. The availability of data at the level of farming households and communities is low in the developing world compared to the developed world.

These sources have led to much data being potentially available for research. However, these data are often closed, being available only for specific purposes or not well managed for future accessibility. In recent years, the open data movement has been raising the awareness of the value of data and promoting methods of availability, accessibility and provenance. Within this open data movement, governments, international organizations, research institutions, and businesses work to offer open access to their data sets to make re-use easier. This work also requires infrastructure to serve the data, such as data.gov, data.gov.uk, data.overheid.nl, and data.fao.org. Global Open Data for Agricultural and Nutrition (GODAN, *godan.info*) is a particularly relevant initiative for open data in the area of food security; it is a multi-party discussion and advocacy forum initiated by the U.S. and UK governments and supported by many different parties. In science, several specialized journals to publish data files are appearing, for example the Open Data Journal for Agricultural Research (*www.odjar.org*) that originated from AgMIP.

### 4.2 New data sources for agricultural modeling

New data technologies are achieving maturity for use in agricultural systems modeling: mobile technology, crowdsourcing, and remote sensing. The emergence of mobile technology significantly supports the advancement of crowdsourcing (sometimes called citizen science or civic science, or volunteered geographic information systems).) Mobile phones, GPS, and tablet devices act as sensors or instruments that directly place data online, with accurate location and timing information. These techniques are often seen as an opportunity for *near sensing*: the use of sensor-equipped tools in the field for capturing observations, e.g., temperature measurements on the basis of data derived from a mobile device. There are also special tools such as leaf area index sensors and unmanned aerial vehicles (UAV), which obtain more location-specific data. These crowdsourcing technologies offer the opportunity to gather more data and at lower cost. In these cases, citizens help to collect data through voluntary efforts, for example biodiversity measurements, mapping, and early warning. Smallholders, citizens, and organizations can thus manage their own data as well as contribute to public data. This offers many possibilities (especially as the technology is still in its infancy), with some successful applications such as IIASA's Geowiki (*geo-wiki.org*). Crowdsourcing is sometimes organized as public events, for example, air quality measurements in the Netherlands on the same day at the same time (Zeegers, 2012).

Earth observation through satellites now provides a continuous record since the early 1980s, forming a data source for time-series observations at any location. More detailed satellite data are coming online through NASA and EU space programs. This leads to an increasing demand for satellite-borne data analyses and applications.

### 4.3 Data quality and interoperability

Making data available to models requires processing (geo-spatial and temporal) and transformation (aggregation, completeness checking, semantic alignment) to ensure quality (provenance, ownership, units), consistency (resolution, legacy), and compatibility with other sources. For most current integrated modeling approaches, these steps are done in a semi-automated way and on an ad hoc basis for each modeling project. Laniak et al. (2013) report that solutions to this issue are beginning to emerge including the GeoSciences Network (GEON, Ludäscher et al., 2003), Data for Environmental Modeling (D4EM, Johnston et al., 2011), and the Consortium of University for the Advancement of Hydrologic Science (CUAHSI: Maidment et al., 2009). With increased data availability, needs grow to ensure interoperability by aligning both syntax (formats) and semantics (definitions) (Antoniou and van Harmelen, 2004). Improved data interoperability creates new opportunities for all types of analysis and the development of new products. However, the necessary standardization has not yet been reached. There are technical standards that are maintained by the International Organization for Standardization (ISO), W3C (World Wide Web Consortium), and Open Geospatial Consortium (OGC), which, however, do not cover connection to the content level of the significance and usefulness of the data. To this end, there are several developments around semantics, which are intended to lead to better descriptions of concepts and relationships between concepts in data sources: AGROVOC from FAO, CABI's Thesaurus, and the CGIAR crop ontology. These efforts have limited practical application for modeling because quantitative data are not effectively treated. For example, ontologies and thesauri rarely include a specification of units. Efforts have been made to fill this gap: Janssen et al., 2009, Fig. 4 combined vocabularies from different agricultural domains, thus facilitating linkage between crop and economic models. The ICASA data standard for cropping systems modeling (White et al., 2013) was re-used in the AgMIP data interoperability tools (Porter et al., 2014) as a means of harmonizing data from various sources for use in multiple crop models and other types of quantitative analyses. Such efforts are, however, still in their infancy (for a discussion see Athanasiadis, 2015), and will benefit from a merging of data translation tools with semantic ontologies for wider discovery of data and tools (Lokers et al., 2016).

**Fig. 4:**
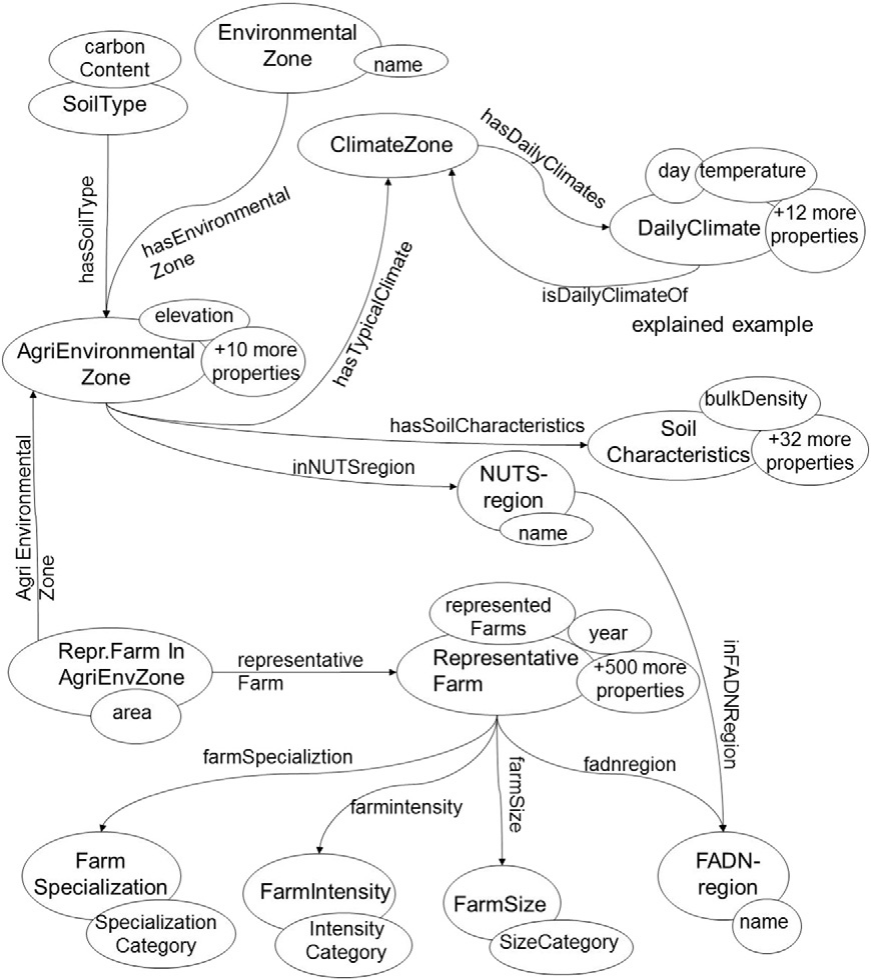
A concept-relationship diagram representing relationships between farms, climate and soil information for Europe, based on Janssen et al. (2009).

### 4.4 Openness, confidentiality, privacy, and intellectual property issues

A major motivation for NextGen agricultural systems modeling is to open up access to data and software that have previously been inaccessible for various reasons, in ways that facilitate discovery, composition, and application by a wide variety of researchers and disciplines. However, while NextGen will certainly benefit from a growing interest in open data and open source software, confidentiality, privacy, and intellectual property issues remain important. Openness and confidentiality (and related topics) are interconnected (see Antle et al. (2017) for more discussion on cooperation models).

Openness refers to facilitating access to data and software. This entails appropriate licensing schemes being endorsed to allow access to information. While reproducibility of science has always been advocated, the typical interpretation in natural sciences does not include providing access to data sources and software as a default position. The reasons for this vary in different parts of the world, but include most notably lack of legislation that obliges public access to data, limited credit to authors who release datasets or software, a lack of sustainable funding mechanisms for long-term collection and curation of important classes of data, and technical difficulties in managing and sharing data.

These obstacles can be overcome: there has been significant progress in promoting open access to data in other disciplines, as evidenced by collections such as the iPlant collaboration for genetics data (*iplantcollaborative.org*), the National Ecological Observatory Network (*neonscience.org*) for environmental science observations, and the Earth System Grid Federation (earthsystemgrid.org) for climate simulation data (Williams et al., 2009).

One important aspect is that licensing arrangements should facilitate ease of access. In our view, simple open-access licenses that give clear rights and obligations to the users of data need to be endorsed for use in agricultural modeling. In other disciplines, scientists have struggled to cope with complex licenses that ultimately are introduced only for enforcing certain wishes of the data owners, and that hinder those who try to reuse the dataset. For the purposes of NextGen agricultural knowledge products, data (and software) licenses need to be (i) as simple as possible; (ii) compatible with one another (as in the Creative Commons); and (iii) non-invasive, i.e. they should not oblige licensees to pass all licence conditions on to their users. These attributes that support openness will need to be balanced with a range of levels of permit control over access to data and software (e.g. to require attribution). Many kinds of data that are critical to NextGen (for example weather records) are curated on behalf of many users by distinct communities of practice. In these situations, NextGen will need to have an influencing rather than a leading role in structuring data-licensing arrangements. This is not the case for data collected primarily in the process of agricultural production such as management practices, yields or cultivars. There NextGen community needs to actively shape a licensing environment for this information that facilitates sharing and reuse.

Privacy and confidentiality issues arise in several regards. Agricultural data may come packaged with sensitive or confidential information: for example, nominal records, geographic location, economic information, and agronomic practices can reveal a subject's identity, location, or entrepreneurial knowledge. While such information can be protected by not disclosing it, data anonymization or obfuscation mechanisms are sometimes needed to permit disclosure while protecting privacy (CAIDA, 2010). There are also techniques for ensuring privacy preservation during computations (e.g., see Drosatos et al., 2014). The NextGen community needs to invest in protocols for protecting privacy and confidentiality.

## 5 Visualizing and interpreting data and model outputs

### 5.1 Traditional visualizations

Tools to enable visualization of agricultural source data, model outputs and synthesized data products are used to enhance the discovery and understanding of information for all users, including data collectors, model developers, model users, integrative research, application developers, and end-users. To make sense of large amounts of unfamiliar or complex data, humans need overviews, summaries, and the capability to identify patterns and discover emergent phenomena, empirical models, and theories related to the data (Fekete, 2013).

Currently most visualization in agricultural systems modeling is organized in an ad-hoc way. Visualization modules are added to models to produce graphs, tables and maps, or else model outputs are transferred to other packages (typically spreadsheet or statistical programs) for analysis. In these types of packages, visualizations are prepared as messages for scientific papers. In cases where models are applied on a more regular basis for a specific purpose, more elaborated visualizations have been built, for example for Monitoring Agricultural Resources (MARS) Unit of the European Union (https://ec.europa.eu/jrc/en/mars) or FEWS-NET (www.fews.net), which account for specific user needs, going beyond those of scientific visualizations.

### 5.2 Visual analytics and big data

A major challenge for NextGen is to include data visualization tools that support the routine exploration of, and interaction with, big data. The traditional workflow of loading a file, processing it, and computing some analytical quantities may not work well when exploring large datasets. The analyst may need to try several processes and methods in order to find relevant results. With big data, a loose coupling between visualization and analysis presents problems, as data transfer time can exceed the time used for data processing. Visual analytics is a branch of computer science that blends analytical algorithms with data management, visualization, and interactive visual interfaces. Visual analytics tools and techniques are used to synthesize information from massive, frequently updated, ambiguous and often conflicting data; to detect the expected and discover the unexpected; to provide timely, defensible and understandable assessments; and to communicate those assessments effectively (Thomas and Cook, 2005). Visual analytics consists of algorithms, representations, and big data management. Currently operationally used state-of-the-art analytics and data management do not yet meet the requirements for big data visual analytics (Fekete, 2013), as they cannot yet process enough data rapidly or in a suitable way to process them and help in obtaining understanding. Data management and analysis tools have begun to converge through the use of multiple technologies, including grids, cloud computing, distributed computing and general-purpose graphics processing units. However, visualization has not adequately been taken into account in these new infrastructures. New developments in both data management and analysis computation will be required to incorporate visual analytic tools into these infrastructures (Fekete, 2013, Fekete and Silva, 2012).

High Performance Computing (HPC) has been used to accelerate the analytics of big data, but for data exploration purposes, data throughput may limit its usefulness. Implementations of existing algorithms such as hierarchical clustering and principal component analysis have been used to pre-process data. New types of workflows are being developed for use in visual analytics, including reactive workflows (e.g., EdiFlow, Manolescu et al., 2009), which specify that a set of operations occur each time the data change, and interactive workflows (e.g., VisTrails, Callahan et al., 2006), which interactively build and run a sequence including visualizations. VisTrails tracks workflow changes to create a provenance record for visual outputs.

## 6 Modeling concepts and methods of model development

### 6.1 Model creation, composition and reuse

Modeling of agricultural systems is influenced simultaneously by the creators' scientific viewpoints and institutional settings, and by differing views on the relationship between models and software. Alternative perspectives in each of these domains emerged in the early days of the discipline and persist to this day. For example, in cropping systems modeling, the physiologically-driven, bottom-up scientific strategy of orderly generalization that is discernable in the Wageningen group of crop models (van Ittersum et al., 2003) contrasts with a more top-down, ecosystem-oriented perspective exemplified by the SPUR rangeland model (Wight and Skiles, 1987). These different perspectives result in different choices about the detail with which biophysical processes are represented. Even when working from a similar scientific perspective, a scientist who constructs a model as a single individual (for example in a PhD dissertation: Noble, 1975) will follow a different process of model specification and implementation from that used by a large team working in a formally managed project (e.g., the Ecosystem Level Model: ELM, Innis, 1978). Researchers who view a model as primarily a mathematical system tend to implement them within generic computational packages such as ACSL (Mitchell and Gauthier, 1976), CSMP (the Continuous System Modeling Program) or Simile (Muetzelfeldt and Massheder, 2003), in which the model proper is a document. In contrast, researchers for whom models are engineering artifacts tend to implement them as stand-alone programs (e.g., CERES-Maize, Ritchie et al., 1991), or as part of modeling frameworks.

### 6.2 Modularity, components and “plug-and-play” approaches

Over time, bottom-up models at lower spatial scales have expanded their scope and models at higher spatial scales have included greater detail. One consequence has been a clear trend towards modularization of models, in terms of both concepts (Reynolds and Acock, 1997) and the way they are coded. In a continuation of this trend, several modeling disciplines have adopted a modular approach to constructing particular simulations as well as the models on which simulations are based, i.e., modularity in the configuration of simulations (for example, Jones et al., 2003, Keating et al., 2003, Donatelli et al., 2014, Janssen et al., 2010). The rationale for this approach to model development is threefold:

(i) to allow model users to configure simulations containing alternative formulations of biophysical processes, based on the need for a particular level of detail or else to compare alternative sub-models;

(ii) to permit specialists in particular scientific disciplines to take on custodianship of sub-models, while ensuring that the larger systems model remains coherent; and

(iii) to minimize, and facilitate easier diagnosis of, unexpected consequences when a sub-model is changed.

In practice, encapsulation of sub-model logic in components needs to be accompanied by transparency through adequate documentation and/or open source implementations, if the confidence of model users is to be maintained; black box sub-models are less likely to be trusted.

As the number of components has increased, it has become natural to assemble them together. While composing large models this way seems both natural and trivial, this is not the case. New limitations are introduced when a model is encoded in a programming language, and seldom are these assumptions represented in the model design or implementation (Athanasiadis and Villa, 2013). Models, as implemented in software, do not usually declare their dependencies or assumptions and leave the burden of integration to modelers. This situation has been the driving force behind many efforts focused on automating integration by providing modeling frameworks (i.e. computerized e-science tools for managing data and software) so as to assist scientists with the technical linking of models to create scientific workflows. A modeling framework is a set of software libraries, classes, and components, which can be assembled by a software developer to deliver a range of applications that use mathematical models to perform complex analysis and prognosis tasks (Rizzoli et al., 2008). Modeling frameworks claim to be domain-independent; however, many of them originate from a certain discipline that drives several of their requirements. In agro-ecosystem modeling, several frameworks have been developed and used by different research groups, such as ModCom (Hillyer et al., 2003), the Common Modeling Protocol, (Moore et al., 2007), BIOMA (Donatelli and Rizzoli, 2008), and the Galaxy-based FACE-IT (Montella et al., 2015). No consensus on how to implement component-level modularity in agro-ecosystem models can be expected in the near future (Holzworth et al., 2015). While the various frameworks show a strong family resemblance, the differences between them, which reflect different points of departure on the mathematics-to-engineering spectrum and also different views on the trade-offs involved in decentralizing model development, mean that the technical barriers to linking them together are quite high. Arguably mosdt framework development has occurred within disciplines in linking models together in operational model chains (see Holzworth et al., 2015 for a discussion of these developments in cropping systems models), while truly interdisciplinary modeling has only been achieved occasionally and hardly at all with rigorous scientific methods. Thus, a methodological and conceptual challenge for a NextGen modeling community is to extend the lessons learnt in building modeling frameworks for specific domains so that inter-disciplinary modeling analyses can be constructed that that produce robust and defensible results, are calibrated with observations, are transparent in methods and calculations, and are useful for answering scientific or policy questions.

Experience from SEAMLESS (Janssen et al., 2011), the AgMIP model intercomparisons (Asseng et al., 2013, Bassu et al., 2014), and pSIMS (Elliott et al., 2014) suggests that even when a rigorous modeling framework is used, a considerable amount of software for converting and translating data between different units, formats, grids, and resolutions needs to be written. In some cases, translation tools are required to integrate data sources and models that do not adhere to common standards. In other cases, translation tools are required because different communities adhere to different standards.

Lloyd et al. (2011) compared four modeling frameworks^1^1 for implementing the same model. They investigated modeling framework invasiveness (i.e. the amount of change required in model code to accommodate a framework), and observed (i) a five-fold variation in model size and (ii) a three-fold variation in framework-specific function usage compared to total code size. These findings imply that there is a large impact of the framework-specific requirements on model implementation, and that lightweight frameworks do indeed have a smaller footprint. Despite the advantages that modeling frameworks were supposed to deliver in easing software development, they are mostly used within the groups that originally developed them, with little reuse of models developed by other researchers (Rizzoli et al., 2008). At the same time, modeling software reuse is hindered by other issues such as model granularity.

### 6.3 Model granularity

The goal of software for integrated modeling is to ensure soundness of results and to maximize model reuse. This can be achieved by finding the right balance between the invasiveness of the modeling framework, as measured by the amount of code change to a model component required to include it in a framework, and the expected benefit of component reuse. A key factor in this balance is the granularity (i.e., the extent to which a larger entity must be decomposed into smaller parts) of the model components. The choice of module granularity involves setting the boundary between one model or sub-model and the next, which can be a subjective and subtle process (Holzworth et al., 2010). Also, if a modeling framework is to support a range of different process representations of differing complexity (for example sub-models for multi-species radiation interception), then data structures and software interfaces need to be carefully designed to be both highly generic in the way they describe the relevant features of the system, and also to have unambiguous semantics. This design work, which is essentially a form of conceptual modeling, can improve the clarity of scientific understanding of ecosystems, but it is unavoidably time-consuming and has been considered an overhead by most modelers either developing their own components or using components developed by others. Most currently used agricultural models tend to have their subcomponents tightly coupled (possibly for better performance), which makes component substitution a laborious task that needs heavy code disaggregation and restructuring, while additional calibration may be needed.

Highly disaggregated components increase the complexity of finding sub-models that are compatible with both the software interfaces and the scientific requirements of a model. The number of connections increases with finer granularity. In contrast, larger, more complex sub-modules reduce the probability that a re-user can find a suitable component due to the more complex interface. Integrated model calibration and validation, in addition to unit testing of individual components, is an important aspect of any modeling system, but becomes even more critical as components are shared and can be pulled from a wider selection. So far there are no good documented examples of component re-use over modeling frameworks indicating that this has not happened much so far. There needs to be a phase a trial and error of incorporating components across modeling frameworks, after which best practices for granularity can be defined for component design.

### 6.4 Process of model development

Most of the collaborative development methodologies have been developed by the open-source movement. Open-source methods are not a prerequisite for collaborative development, however; many closed-source products follow similar methods for project management. The seminal work of Raymond (1999) introduced two major project governance models, the Cathedral and the Bazaar, that still dominate software development in various ways. In the Cathedral model, code is shared only among the members of the development group, and decisions are taken through a strict hierarchy of roles. In contrast, the Bazaar model allows a large pool of developers to contribute with improvements, changes and extensions over the Internet. In the development of agricultural models, almost solely Cathedral models of development have been used with one custodian managing all the code.

Where a single organization or a small group of individuals takes responsibility for specifying the design of a modeling system, then the simplest method of collaborative development is a Cathedral approach. The main benefit of the approach is that there is a clear definition of what constitutes a given model at a given time. However, Cathedral approaches to collaboration are unlikely to be workable for many elements of a NextGen agricultural modeling system; there will simply be too many peer stakeholders. The ‘Bazaar’ alternative approach to collaboration introduces the use of a common code repository with a version control system together with social technologies to manage modifications.

Collaborative approaches to model and application development are a consequence of open-source development and carry transaction costs such as meetings, increased communication, and increased effort in documentation and quality control; and benefits that include better transparency, lowering the barrier to new contributors, and peer-reviewing of design and code implementation. The costs to the modeling community of maintaining software quality assurance technologies and governance mechanisms are non-trivial; but the costs to a model developer of joining an open-source community (to translate existing code or adjust to a different conceptual framework) can also be significant. Most important is the cost of paradigm shift. With respect to NextGen models, creating an open-source community around a model will not be as straightforward as converting an existing individual model to open source. In merging different communities and scientific approaches, a medium-to-long-term investment by a core group of adopters is vital to achieve the critical mass of benefits that are required to make participation attractive.

## 7 Infrastructure and interfaces

### 7.1 Interfaces for end users

Much consumer and business software today is not installed on PCs but is instead delivered by cloud-hosted services accessed over the network from a Web browser, often running on a mobile phone or tablet: this approach is called software as a service (SaaS: Dubey and Wagle, 2007). Intuitive Web 2.0 interfaces make user manuals largely a thing of the past. The result of these developments is a considerable reduction in operating cost, often an increase in usability, and above all a dramatic increase in the number of people who are involved to design, deliver and maintain these systems. The technology for delivering information through mobile and other devices has rapidly developed in a short time. This does not mean that agricultural models were extensively adopted these developments, as they seem not to be ready to do so in terms of their design, flexibility and ease of deployment. Many of agricultural models and their interfaces have been developed with the researcher as user in mind and are based on Microsoft Windows-based PC's, as the start of their design was often more than 20 years ago. Many models have yet to be rigorously improved to adhere to SaaS principles with respect to data, licensing, model design and granularity and general robustness of application to allow them to be linked in operational tools for end-users.

### 7.2 Data and model discovery

Use of big data, component-based models, synthesized information products, and apps for delivering knowledge through mobile devices can all be easily envisioned using technologies that exist today. A grand challenge for the NextGen agricultural applications will be to provide common protocols for making these numerous databases, models, and software applications discoverable and available to users and developers through web services and distributed modeling frameworks. By using common semantic and ontological properties in Web 3.0 interfaces, the data and modeling components can be made available for coherent use in a proposed NextGen platform. Web 3.0, also called the Semantic Web, refers to standards being developed by the World Wide Web Consortium (W3C) for data discovery, sharing, and reuse across applications, enterprises and community boundaries (*www.w3c.org*). Linked data refers to connections between the contents of datasets to build a “web of data.” This technology is relatively new and as yet unproven for practical use in the scientific and big data realms (Janssen et al., 2015). Many claims have been made regarding the potential of linked open data using W3C protocols, but it is not yet clear whether the complexity and cost of designing these systems is worth the benefits. Tools for working with linked data are not yet easy to use and few people have access to the technology and skills to publish linked datasets (Powell et al., 2012). However, despite the lack of maturity of this technology, it holds great promise for use in a distributed ICT modeling framework, provided that the Web 3.0 protocols continue to develop and coalesce around common standards and that tools are introduced that allow more rapid development of customized and complementary ontologies.

Other elements of a distributed modeling framework, such as cloud and web-based computing, movement of big data across the web, and software-as-a-service (SaaS) are already in wide use. For example, SaaS is being used to deliver research data management (Foster, 2011), data publication (Chard et al., 2015), agricultural modeling (Montella et al., 2015), genome analysis (Goecks et al., 2010), and plant modeling (Goff et al., 2011) capabilities to researchers. Commercial cloud offerings such as those provided by Amazon Web Services, Google, and Microsoft Azure provide both on-demand computing and storage and powerful platform services. Standards have been established for many aspects of cloud computing (e.g., the Open Cloud Computing Interface and Cloud Data Management Interface). However, standardization gaps still exist for many other areas as delineated by the National Institute of Standards and Technology (NIST, 2013). These standardization gaps include SaaS interfaces for data and metadata formats to support interoperability and portability and standards for resource description and discovery. The NIST report lists 15 groups that are actively working on development of standards for all aspects of cloud-based computing.

## 8 Conclusions and research agenda

Overall agricultural systems modeling needs to rapidly adopt and absorb state-of-the-art data and ICT technologies with a focus on the needs of beneficiaries and on facilitating those who develop applications of their models. This adoption requires the widespread uptake of a set of **best practices** as standard operating procedures. Focussing on single technologies will be insufficient if agricultural models are to achieve their true potential for societal benefit, simultaneous improvements in practice will be needed across the full range of information & communication technologies and methods addressed in this paper.

First, modeling developers of models and application chains need to follow good coding practices as long established in software engineering. For example, with respect to the modeling itself, clearly separating code that implements model equations from their user interfaces will make constructing computational chains easier. At the same time, coding a model needs to become simpler. By providing appropriate domain-specific structures and functions as libraries, we can enable NextGen model implementations that are significantly smaller, in terms of lines of model-specific code, than today's models. As development and maintenance costs tend to scale with lines of code (Boehm, 1987), such developments will have a considerable positive impact.

Second, interoperability of both data and models has to become the operating standard. All elements of the system should be linked via intuitive, Web 2.0 interfaces, with associated REST (Representational State Transfer) application programming interfaces for programmatic access. This approach will allow for self-documenting applications and interfaces and will facilitate navigation and integration of the various modeling and data components. Not many agricultural models follow this design to date. Software and data should be cloud-hosted or delivered via services to permit access by any authorized user, without the need to install local software.

Third, access to data and software needs to be rapidly improved. Ideally, most data and modeling components will be free in both senses of the word, with little to no cost and few to no restrictions on use, so as to encourage an active community of application developers to provide value-added products to end users The modeling community must stimulate and organize ease of upload and publication of new data, software, and workflows. The principle of “publish then filter” must be followed, to encourage sharing of data and software. Feedback mechanisms (such as ratings and post-publication review) could be provided to identify what is good—rather than interposing onerous curation processes that will inevitably limit data and code sharing. Mechanisms (e.g. digital object identifiers) should be integrated for citing contributions of data and software and for tracking accesses to contributions, in order to provide positive and quantitative feedback to contributors.

Fourth, the data integration challenge needs to be ubiquitously addressed. Data are now siloed in different domain-specific repositories, and interoperability across domains and scales is very weak, slowing the efficiency of analytical solutions that modeling is (Lokers et al., 2016). As a first step, common vocabularies and ontologies need to be constructed to allow for data interchange among disciplines. Development of vocabularies has the important side effect of requiring that the disciplines work in a coordinated way, thus breaching the disciplinary silos that currently impede progress in integrated modeling. Use of linked data protocols will allow interpretation of data from multiple, distributed sources. While an ontological framework may seem an overhead for many researchers, still there is an emergent need for standardization actions for both terminology and data formats (i.e., see Porter et al., 2014, Janssen et al., 2011, Lokers et al., 2016).

Fifth, NextGen agricultural models must be applied to reference data with quality standards, i.e., as those proposed by Kersebaum et al. (2015). Such reference data sets for applications (at different scales, domains and purposes) need to be defined and published as open access, so that new implementations can be tested and benchmarked easily.

Sixth and last, user requirements of beneficiaries benefiting from applications of agricultural systems models need to be investigated using state-of-the-art software design techniques. This could enable the development of a suite of output-presentation components that are in the public domain, well-designed for agricultural problems, and suitable for use across many different applications.

In conclusion, as an overall research challenge, the interoperability of data sources, modular granular models, reference data sets for applications and specific user requirements analysis methodologies need to be addressed to allow agricultural modeling to enter in the big data era. This will enable much higher analytical capacities and the integrated use of new data sources. We believe that a NextGen agricultural modeling community that follows these good practices and addresses the research agenda is likely to gain a substantial following and to spur increased collaboration within and between communities. We expect it to provide significantly enhanced tools to help deliver sustainable food production under both today's and tomorrow's changing climate conditions.

## Appendix A Supplementary data

Supplementary material. Image 1

## Footnotes

1 The four frameworks were the Earth System Modeling Framework (ESMF), the Common Component Architecture (CCA), the Open Modeling Interface (OpenMI), and the Object Modeling System (OMS).
