# Supplementary material for: Towards a new generation of agricultural system data, models and knowledge products: Information and communication technology
Source: Agric Syst. 2017 Jul;155:200–12. doi: 10.1016/j.agsy.2016.09.017 (PMC5485661; doi:10.1016/j.agsy.2016.09.017)
Supplement: Supplementary file 1 — Supplementary material. [file mmc1.docx]

# Supplementary Material: User scenarios for reference use cases 2 to 5

## Use Case 2 - Developing and evaluating technologies for sustainable intensification

### Problem statement

Debora is a plant breeder/geneticist at CIMMYT, the International Center for Maize and Wheat Improvement, working on developing a drought- and heat-tolerant hybrid of maize. She wants to evaluate the potential adoption and impact of maize varieties with particular characteristics across the widely varying conditions in Africa, where maize is an important crop. She realizes, however, that maize is only one part of the complex farming systems used by most farmers, which typically involve multiple crops and livestock. Thus, rather than evaluating maize by itself as had been typically done by most research programs, she wants to evaluate the potential of new varieties within these complex systems. Moreover, she wants to know whether new varieties meet goals for sustainable intensification, such as improving productivity—not just in the short term, but also when longer-term impacts on soils, water, and greenhouse gases are taken into account.

### Current deficiencies

The analyses that Debora wants to perform require access to a database of farming systems typologies containing information about the types, management regimens, and frequencies of occurrence of crop and livestock production systems in the region—a database that does not currently exist. A crop variety library linked to both genetic markers and crop modeling parameters for multiple models is also needed, so that Debora can create model parameters using the genetic characteristics of new varieties. So too are regionalized soil and weather databases, with data suitable to drive these models. A wide range of models are required, including mixed cropping-systems models integrated with livestock systems and household economic models, to generate predictions of production and income over a region or country. If Debora wants to estimate the uncertainty of the model predictions, she may choose to run a multiple models under a range of conditions.

Current infrastructures are inadequate to rapidly and accurately address this use case. Gene-based modeling techniques exist (Hoogenboom and White 2003; Messina et al. 2006; Hammer et al. 2006) are progressing rapidly and may be adequate already. However the farming systems topology database and cultivar libraries described in the use case do not currently exist. Existing soils and weather data suitable for regional analyses may be available, but are often not usable directly by crop models due to missing data and incompatible formats. Linking models to perform the uncertainty calculations described in the use case is exceptionally difficult.

### Initial draft of a NextGen application chain

Working with a team of colleagues at her research institution, Debora uses the NextGen Technology Adoption and Impact Tool (TA&IT) for this purpose. This tool integrates the genetic characteristics of the maize varieties with soil, weather, economic, and social data representing the farm populations where the new varieties could be used. Jan’s recent contributions to these databases have been used to update them seamlessly. The research team then simulates the potential for adoption and impacts of the new varieties, providing Debora with guidance for the kinds of genetic modifications that would be most valuable to farmers, and also provide an assessment of the long-term sustainability of the systems.

Figure 5 and the following design-time narrative explore the possible components of a solution for Deborah’s use case.

- Debora sees herself as a “crop breeder, not a modeler” so she engages with a colleague (Eduardo) who has previous experience with the **Technology Adoption and Impact Tool** (TA&IT). Like the models it uses, the TA&IT is more a collection of workflows and components than a single piece of software.
- Eduardo scopes out the analysis requirements with Deborah. By using the query tool associated with the **Global Farming Systems Typology** (GFST) database, they confirm Eduardo’s initial judgment that a farming systems typology at an intermediate level of resolution will provide an appropriate balance between detail and computational effort. Consistent descriptions of approximately 200 **representative farming systems** (RFSs) across sub-Saharan Africa that depend on maize production are selected, along with stored estimates of the populations they support
- Fortunately for Debora & Eduardo, a previous project on conservation farming practices has developed and **recorded templates** (instructions) for translating GFST descriptions into formats needed by several **biophysical models** of smallholder farming systems. The biophysical model configurations include multiple crops, livestock, soil C, N & P cycling and representative diversity of soil types, plus responsive land use and tactical management systems that reflect households’ human capacity, and household consumption of product. They are parameterized from the GFST.
- Eduardo adjusts the templates to place more emphasis on in-crop management of maize, and swaps in a livestock production model that will respond to quality changes in corn residues fed to animals. He chooses the latter from a **library of several livestock production models** that share a common within-simulation interface.
- Debora has seen the results of model intercomparisons and wants to be reasonably sure the analysis isn’t dependent on the choice of maize model. Eduardo therefore builds three versions of the mixed farming model, using **3 different maize growth models**. To include the third maize model, he has to write a small **OpenMI translator widget** but, working from the example already available for maize model #1, this only takes a day or two. Eduardo adds this work to the ever-growing interface repository.
- Meanwhile, Debora and the crop physiologist in her team have been describing the target traits that are to be evaluated in terms of their physiological function. Eduardo then arranges teleconferences with Fritz, Gemma, and Hortense, who are expert users/developers of each of the maize models. At these meetings the best ways to describe each trait in terms of parameters of each model are decided.
- Standard **translator Web services** are built into the **modeling workflow**, so that simulations can access weather and soils data for each RFS model from consistent global databases and convert them into formats that are useable by the models.
- A set of preliminary runs are carried out using current maize genetics and are checked (using standardized reports) to ensure that inter-annual distributions of maize production, other crop and livestock production, cash and labor budgets are sensible. This step goes smoothly because these “base” models are being re-used.
- Output from the biophysical models, along with RFS-specific context, are piped to **cash and labor budgeting modules**, an **assessment of** changes in the adequacy of **household diets and behavioral information about adoption**, and a semi-quantitative tool that accounts for factors (cost, complexity, etc.) that will influence the **adoption** of new genotypes.
- Eduardo advises Debora on the design of a simulation experiment that varies each target genetic trait against the existing genetic background and management system of each RFS. They also decide to include **systematic variations in fertilizer input intensity** and the **proportion of land devoted to maize**, in case Genetics x Management x Environment (GxMxE) interactions are important.
- Because the standard TA&IT “trait evaluation” workflow is being adapted to this analysis, Eduardo has a straightforward job of **synthesis** once the analysis is run. A **collection of useful statistics and presentations** has already been prepared (adoption rates, maps of where the net benefits are highest, differentials in effect on richer and poorer household types, income-risk-natural resource management tradeoffs) and the TA&IT-specific visualization tool allows him to show the results to Debora for her interpretation.

***Figure 5: the components of the data, modeling and delivery infrastructure according to application chains to deliver use case 2***

## Use Case 3 - Investing in agricultural development projects that support sustainable intensification

### Problem statement

Stanley is an investment manager for a prominent foundation, and he needs to evaluate a project proposal for small farms in Kenya that will increase the intensity of production by increasing fertilizer use per hectare on cash crops while maintaining the current sustainable nutrient balance between pasture grasses, crop residues and animal manure. Before authorizing a project that combines extension information and fertilizer subsidies, Stanley wants to evaluate whether the higher crop yields would induce a non-sustainable system once the initial period of fertilizer subsidies and extension was completed.

### Current deficiencies

In current agricultural systems modeling, many models (i.e., livestock, crop production, economics, soil, water) would need to be linked and different pieces of information extracted from them, and then combined to produce relevant indicators, mostly done by the researcher or analyst personally, or even a combination of researchers from different domains, with fragmented access to data. Data sources used, their aggregations, and their availability would be managed by each modeler for each model, which creates a confused situation in that the same data are used for different models, but with different formats. A significant coordination effort is required to succeed at all, which focuses on the different modelers applying the models, getting all the relevant data, and ensuring some consistency and interchangeability across model applications.

### Initial draft of a NextGen application chain

Stanley implements the NextGen Project Assessor Tool (PAT) to access data and crop and livestock model components to assess the yield and labor impacts of increased yields. An economic assessment model is used to estimate if the current cropping balance will change under the new fertilizer program and if increased fertilizer costs can be more than compensated by increase in cash crop yields in the long run. A long-term farm level nutrient balance under increased intensification will show whether the new system is sustainable. Stanley would like to evaluate these results under a range of assumptions, and present these to local decision makers so that they share common expectations and uncertainties. For this he uses the NextGen Project Assessor, which opens as a web page on his computer, and he sets up a new assessment, enters data supplied with the project proposal, and links this to general data layers available in the tool. The NextGen Project Assessor uses multiple NextGen Models and the Global Farming Systems Typology as tools for impact assessments. Figure 6 illustrates the components of the proposed solution, which includes the steps listed below.

Ideally in the NextGen modeling framework the necessary data would be available on a common platform in the NextGen Project Assessor Tool (PAT). Data (all formatted to a common data dictionary/ontology) include traditional sources like **household surveys**, **field experiments**, **regional statistics**, but also **crowdsourced recent estimates** of biomass growth and disease spread and **remotely-sensed images** of field distribution and water availability.

**Data assimilation** through models could subsequently be executed with summary processes described in the NextGen Project Assessor library, or by an export to more comprehensive modeling tools following standardized export formats.

Ultimately, in the NextGen Project Assessor, a library of tools can be used to present the best combination of **integrated indicators**, describing the likely impact of the measures proposed in the project in an appealing way for external stakeholders building on state-of-the-art visualization software.

Here it is unlikely that Stanley does all these analyses himself, but instead he invites an analyst to do this for him, once he has formulated his question in the start page of the NextGen Project Assessor.

Crucial to success of the NextGen Project Assessor are: (1) ample availability of good quality data that can be used freely for analysis; (2) easy integration across domains of data and analysis tools without excess in details of any particular domain; and (3) flexibility in import and export of data according to standardized formats to facilitate sharing and visualization. From an ICT point of view, this would require innovations in: (a) data discovery with a necessity to make data searchable and easily transformable, even if residing physically at many different locations across the globe; and (b) easy mash-up of data from different sources using standardized analysis tools in a web-based platform accessible to many different users with different roles with a strong presentation layer.

***Figure 6: the components of the data, modeling and delivery infrastructure according to application chains to deliver use case 3***

## Use Case 4 - Management support for precision agriculture

### Problem statement

Greg is a farmer in the US, with a large corn/soybean-based farm and a high level of mechanization fully equipped with auto-tracking system and high-resolution differential GPS. Greg has a historical archive with more than 15 years of data on crop yield spatial variation at five-square-meter resolution. His tractors are equipped with on-the-go sensors for variable applications of seeding, fertilizer, pesticide, and herbicide. Greg consults with a precision agriculture consultant, Harold, who provides him with up-to-date management prescriptions, tailored to the current crop stage and soil variations in his fields. These strategic and tactical crop management recommendations include variable rate prescriptions for fertilizer/pesticide/herbicide application and accurate harvest recommendations that are automatically integrated in Greg’s Controller Area Network (CAN)-bus enabled tractor for variable rate application of inputs.

### Current deficiencies

Much of the information that Greg and Harold need to realize this use case is available with current technologies, including remote sensing through high-resolution satellites, airborne imagery and drones; farm equipment designed for precision agriculture; and existing crop modeling and data analysis software. What is currently lacking is the ability to link remotely sensed data with a decision support system that allows a farmer to make informed decisions regarding management required for specific locations in a field or farm, translated to the on-farm GIS-equipped machinery. Crop models allow useful extrapolation and prediction for prescriptive management, but most current crop models lack the ability to handle spatially connected processes (i.e., water flow, weeds, and pest dynamics) within a field or landscape. Use of the models with real-time, remotely sensed data is not currently available to farmers or farm advisors.

### Initial draft of a NextGen application chain

Greg receives weekly updates on his smart-phone and tablet from Harold’s Precision Agriculture Company about the status of his crops. Information contained in these updates is obtained from drone flights and crop model predictions using a combination of observed and forecasted weather. Harold’s analysis relies on the NextGen models that are able to deliver strategic and tactical crop management recommendations, process-based variable-rate prescriptions for fertilizer/pesticide/herbicide applications, and accurate recommendations on harvest management. The variable-rate prescription map created by Harold’s company is cloud-based and is automatically integrated in Greg’s CAN-bus enabled tractor for variable rate application of inputs. Greg’s farm technologies allow him to trace back all the activities performed in the field and the harvested product.

The following design-time narrative and Figure 7 describe the components of the data, modeling, and delivery infrastructure that could allow Greg to implement GIS-based precision farm management.

Harold’s Precision Agriculture (HPA) consulting business maintains **high-quality, high-resolution soil attribute maps** for Greg’s fields. These data are considered to be **proprietary**, as Harold and Greg have invested in the collection of the data specific to these fields. NextGen **soil data harmonization tools** were used to prepare the data in a format that can be used by multiple crop models. Soils are tested annually at several locations in the field and the files are updated as newer or corrected data become available and data are stored on a cloud-based server.

HPA buys **high-quality observed weather data** from a service that also prepares **ensembles of seasonal forecast weather data**. These data are combined with **rainfall data** recorded in Greg’s fields. These cleaned and combined data are also provided in a **harmonized format** ready for use by crop models. HPA serves the observed weather data on public servers as **linked open data**, which are then available for discovery and use by other NextGen models. The ensembles of weather forecasts are proprietary, and although stored in the same formats, are not made available as open data.

HPA owns several drones, which are flown over Greg’s fields bi-weekly to generate precision maps of leaf area index, biomass and chlorophyll content, which can be converted into site-specific nitrogen uptake by crops. A **history of** these **aerial field maps** can be retrieved to generate time series for the crop growth at any point in the field.

Greg’s CAN-bus enabled tractor allows Greg and Harold to archive all precision management data for the variable rate application of inputs to Greg’s fields. HPA maintains **proprietary software** that converts these management data to crop model-ready formats.

HPA’s crop modeling staff have pre-configured the NextGen **Precision Agricultural Management Tool** (PAM-Tool) to generate an ensemble of crop growth simulations for Greg’s fields using the detailed soil maps, observed weather data, ensembles of seasonal weather forecasts, cultivars planted and management history. A **data assimilation package** is included in the crop model used for simulation so that estimations of in-season biomass and LAI can be improved using Bayesian filtering techniques.

The PAM-Tool working on a **high-performance cluster** quickly identifies optimum irrigation scheduling; fertilizer, pesticide and herbicide applications; and harvesting schedules. These recommendations are sent via smartphone for review by Greg and Harold. If approved, the recommendations are sent to the tractor’s computer for implementation.

Greg’s use of the PAM-Tool relies heavily on his soil attribute maps. These are derived from his proprietary data using inference algorithms that were created by publically-funded research and are made freely available. As matter of enlightened self-interest and of citizenship, Greg agrees to a request to make his soil attribute data available (via a standard soils description Web service) for the quarterly re-estimation of the empirical parameters used by these algorithms.

***Figure 7: the components of the data, modeling and delivery infrastructure according to application chains to deliver use case 4***

## Use Case 5 - Supplying food products that meet corporate sustainability goals

### Problem statement

Jennifer is an economic analyst with in a corporate sustainability group. This group has embarked on efforts to make sustainability the core of their mission: marketing food while conserving resources. She is assessing the lifecycle of food products to find ways to conserve energy, save water, minimize waste, and reduce greenhouse gas emissions in an effort to make these products more sustainable from the farm to fork. Specifically she is looking at the potato chips division as a case, having in mind that the corporation set an ambitious reduction target for greenhouse gas (GHGs) emissions by 2020. She wants a monitoring and evaluation system, in which she can track the different sources of emissions, synthetically test interventions, and follow the year-to-year variability in the emissions.

### Current deficiencies

Current modeling of supply chains mostly uses life cycle analyses that have little connection to landscape or field level modeling. Innovations in modeling are required to bring new algorithms forward; that can also parse data near-real time along the supply chain and that can compute implications in nutritional content of the different foodstuffs. Data from remote sensing, climate scenarios and weather forecasts are not extensively used to predict supply chain implications using state of the art models. Also, the combination of data and information from private large corporations and public sources only occurs on the premises of the private corporation.

### Initial draft of a NextGen application chain

Figure 8 and the following design-time narrative present a proposed solution for Use Case 5 that uses the proposed NextGen **Supply System Assessment Tool** (SSAT). This tool is initiated and implemented by a consortium of large agribusiness companies. In recognition of their common interest in the transparency of their corporate sustainability policies, the consortium places the SSAT code in a public software repository and invite proposals for improvements.

Using a Web service, Jennifer works with her analysis team to access SSAT. This tool monitors and visualizes the GHG emissions in the supply chain at the different steps in the supply chain. To use the tool, Jennifer and her team first configure it with information for their chips **supply chain** with factory locations, approximate location of farmers delivering input for the chips, and transportation moves to and from the different locations in the supply chain.

The tool offers real-time weather and historical climate conditions around the globe as standard information, together with exchange rates, trade flows, soils, population densities, and GDP.

Jennifer discovers that most GHG emissions occur in crop production, so she sets out to identify strategies that will optimize the amount of fertilizer to be used at a particular location with the goal of increasing yield and reducing greenhouse gas emissions.

Through a web service provided by YieldGap.org, she imports data on **yield gaps** for crucial crops in the most promising production regions for her corporation, and estimates the room for improvement in yield, while at the same time getting information on **nitrogen application** **rates** and **irrigation** techniques from the local supply chain contacts and the Global Farming Systems Typology.

With information from remote sensing and seasonal weather forecasts, that produce **yield forecasts**, she designs different management strategies for the current season, with estimates of the expected yields and GHG emissions, also with timing for harvest to optimize transport movements.

For Jennifer, the NextGen Supply System Assessment Tool also computes a total of **GHG emissions** **saved** over the supply chain as an estimate. Proposals for management practices to be included in supply contracts with farmers are presented and discussed with the local supply chain managers of the corporations; the resulting local knowledge is incorporated as changes in the SSAT inputs data and new simulations are carried out before the final supply contracts are prepared.

***Figure 8: the components of the data, modeling and delivery infrastructure according to application chains to deliver use case 5***

# References

Hammer, G., M. Cooper, F.Tardieu, S. Welch, B. Walsh, F. van Eeuwijk, S. Chapman, and D. Podlich. 2006. “Models for navigating biological complexity in breeding improved crop plants.” *Trends in Plant Science* 11(12):587-593.

Hoogenboom, G., and J.W. White. 2003. “Improving Physiological Assumptions Of Simulation Models By Using Gene-Based Approaches.” *Agronomy Journal* 95(1):82-89.

Messina, C. D., J. W. Jones, K. J. Boote, and C. E. Vallejos. 2006. “A gene-based model to simulate soybean development and yield responses to environment.” *Crop Science* 46:456-466.
